# Ferroptosis in liver cancer: a key role of post-translational modifications

**DOI:** 10.3389/fimmu.2024.1375589

**Published:** 2024-04-08

**Authors:** Ying Xu, Zhiyao Xing, Ruaa Abdalla Ibrahim Suliman, Zichuan Liu, Fengyuan Tang

**Affiliations:** ^1^ School of Pharmaceutical Science and Technology, Tianjin University, Tianjin, China; ^2^ Frontiers Science Center for Synthetic Biology and Key Laboratory of Systems Bioengineering (Ministry of Education), Tianjin University, Tianjin, China; ^3^ Thinking Biomed (Beijing) Co., Ltd, Beijing Economic and Technological Development Zone, Beijing, China

**Keywords:** PTMs, HCC, ferroptosis, drug resistance, immunotherapy

## Abstract

Ferroptosis is an emerging form of regulated cell death in an oxidative stress- and iron-dependent manner, primarily induced by the over-production of reactive oxygen species (ROS). Manipulation of ferroptosis has been considered a promising therapeutic approach to inhibit liver tumor growth. Nevertheless, the development of resistance to ferroptosis in liver cancer poses a significant challenge in cancer treatment. Post-translational modifications (PTMs) are crucial enzymatic catalytic reactions that covalently regulate protein conformation, stability and cellular activities. Additionally, PTMs play pivotal roles in various biological processes and divergent programmed cell death, including ferroptosis. Importantly, key PTMs regulators involved in ferroptosis have been identified as potential targets for cancer therapy. PTMs function of two proteins, SLC7A11, GPX4 involved in ferroptosis resistance have been extensively investigated in recent years. This review will summarize the roles of PTMs in ferroptosis-related proteins in hepatocellular carcinoma (HCC) treatment.

## 1 Introduction

Primary liver cancer is a prevalent and serious malignancy, remaining one of the leading causes of cancer-related mortality worldwide (1). The incidence of liver cancer is estimated to increase by 55.0% from 2020 to 2040, with projections of 1.4 million new cases and 1.3 million deaths in 2040 (2). Primary liver cancer encompasses four main types: HCC, Intrahepatic cholangiocarcinoma (ICC), fibrolamellar HCC, and mixed HCC-ICC tumors (3). Among these subtypes, HCC represents the most common form, ranking as the fifth most frequent solid tumor globally and second among all cancers’ death rates worldwide (4). Approximately three-quarters to four-fifths of patients diagnosed with primary liver cancer have HCC. Characterized by rapid progression, high metastasis potential, and late diagnosis at advanced stages, HCC often has a poor prognosis due to high recurrence rates after surgery or other treatments (5).

Besides surgical interventions, such as resection or transplantation for early and/or localized disease, current treatment options include radiation therapy, systemic chemotherapy, and targeted therapies. These targeted therapies include sorafenib, lenvatinib, regorafenib, cabozantinib, and ramucirumab, which target the VEGF receptor-2 signaling pathways (6).

Sorafenib was the first molecularly targeted drug approved for clinical treatment of liver cancer (7, 8). It is a multi-target kinase inhibitor that blocks tumor cell proliferation by inhibiting the activity of RAF-1, B-Raf, and other kinases in the Ras/Raf/MEK/ERK signaling pathways. Additionally, sorafenib inhibits angiogenesis by targeting various receptors, including the hepatocyte cytokine receptor (c-Kit), FMS-like tyrosine kinase (FLT-3), vascular endothelial growth factor receptors (VEGFR-2 and VEGFR-3), platelet-derived growth factor receptor-beta (PDGFR-β), and other tyrosine kinases (9). Administration of sorafenib significantly prolongs median survival time in patients but is often associated with pronounced adverse effects and the emergence of drug resistance (7). Therefore, to investigate the underlying mechanism driving sorafenib resistance and to develop more effective personalized therapeutic strategies represent huge unmet medical needs and clinical challenges.

Lenvatinib was the second agent approved for first-line therapy (10). Lenvatinib, also a TKI, inhibits the activity of several receptors, including vascular endothelial growth factor receptor (VEGFR), fibroblast growth factor receptor (FGFR), rearrangement during transfection of receptor (RET), C-kit, PDGFR-α, and PDGFR-β (11). On August 16, 2018, the FDA approved Lenvatinib, which showed outcomes no worse than Sorafenib, marking the first significant breakthrough in HCC drug treatment in 10 years (12). However, the OS improvement with Lenvatinib was not significant compared to sorafenib (13). Unfortunately, resistance to Lenvatinib is common, underscoring the critical need to understand the mechanisms behind this resistance.

Recently, immune checkpoint inhibitors (ICIs), such as anti-programmed death receptor 1 (PD-1), anti-programmed death ligand 1 (PD-L1), and anti-cytotoxic T lymphocyte antigen-4 (CTLA-4) monoclonal antibodies (Mabs), have been tested in both treatment-experienced and treatment-naive patients. Additionally, combination immunotherapy strategies, including anti-PD-1/PD-L1 monoclonal antibodies with anti-VEGF monoclonal antibodies, TKIs, or anti-CTLA-4 monoclonal antibodies, are being evaluated and continue to be investigated. These approaches aim to expand the response population, overcome resistance, and improve efficacy (14). The combination therapy of atezolizumab (an anti-PDL1 mono­ clonal antibody (mAb)) plus bevacizumab (an anti-vascular endothelial growth factor (VEGF) mAb) represents a new milestone in the field of HCC treatment (15, 16). Nevertheless, the benefits of current therapeutics remain limited, as patients often experience disease recurrence (17).

Cell death plays a pivotal role in the development and pathogenesis of multicellular organisms. In recent decades, various types of cell death have been identified and classified based on their distinct morphological characteristics, biomarkers, or regulatory mechanisms. Notable types include apoptosis, necroptosis, pyroptosis, and autophagy-dependent cell death (18, 19). In 2012, ferroptosis was discovered by Brent R. Stockwell’s lab as a novel form of regulated cell death. This process is characterized by the abnormal metabolism of lipid oxides, catalyzed by iron ions or iron enzymes (20). Distinct from apoptosis, necroptosis, and autophagic cell death, ferroptosis represents a unique, iron-dependent form of intracellular cell death. Morphologically, it is characterized by smaller mitochondria compared to normal cells and increased mitochondrial membrane density (21). Mechanistically, ferroptosis involves the iron-dependent accumulation of lipid peroxidation, leading to cellular demise, and it is regulated by a distinct set of genes including RPL8(ribosomal protein L8), IREB2(iron response element binding protein 2), ATP5G3(ATP synthase F0 complex subunit C3), CS (citrate synthase), TTC35(tetrapeptide repeat domain 35), and ACSF2(Acyl-CoA synthase family member 2) (20). Importantly, ferroptosis can be specifically and efficiently block by Ferrostatin-1, but not ZVAD-FMK or Necrosulfonamide, a potent apoptosis inhibitor and necroptosis, respectively. This indicates that ferroptosis represent a distinct death pathway instead of apoptosis or necroptosis. Moreover, ferroptosis can be potently induced by Erastin, which inhibits glutathione (GSH) synthesis through suppressing SLC7A11 (Solute Carrier Family 7 Member 11, cystine/glutamate antiporter, also commonly known as xCT).

PTMs are chemical modifications of specific amino acids that affect the conformation, activity, interaction, stability, and spatial distribution of most eukaryotic proteins (22). Maintaining proper protein modification homeostasis is crucial for human health. Abnormal PTMs can lead to changes in protein properties and loss of protein function. These changes are closely associated with the development and progression of many diseases (23). The PTM of a variety of proteins (e.g., Phosphorylation, ubiquitination, acetylation, and methylation) also plays an integral regulatory role in ferroptosis (24).

The focus of this article will be on investigating the intricate relationship between ferroptosis, PTMs, and drug resistance in liver cancer.

## 2 Development of therapy resistance and potential mechanisms

Despite significant advancements in tumor treatment, the development of tumor resistance remains a major challenge (25). Resistance to HCC severely impedes the long-term clinical efficacy of existing treatments (26). Therefore, there is an urgent clinical need to overcome drug resistance in refractory HCC.

The mechanism of drug resistance in HCC is a complicated process driven by multiple factors (27). During treatment, tumor cells can develop resistance to chemotherapy, radiation, or immunotherapy. This resistance is driven by various cancer-cell intrinsic factors, including (but not limited to) (i) enhanced expression of specific ATP-binding cassette (ABC) transporters, which can lead to reduced efficacy of anticancer drugs; (ii) increased DNA repair activities for damaged DNA, (iii) heightened tolerance to stressful conditions; (iv) adaptive *de novo* genetic mutations in key cellular pathways and (v) cancer cell undergoing de-differentiation processes and induction of tumor heterogeneities, for instance, mediated by epithelial to mesenchymal transition (EMT) (28). Moreover, the adaptive reshaping of tumor microenvironments adds another layer of complexity to therapy resistance (29, 30).

While sorafenib and lenvatinib appear to be effective in prolonging median survival among HCC patients with limited side effects, adaptive resistance is invariably developed among almost all patients, which subsequently becomes a barrier to extending overall survival rates (31). Hence, understanding the mechanisms underlying therapy resistance is crucial for improving the survival outcomes for HCC patients. Consequently, delineating various cell death pathways in depth will be of great medical interest to overcome therapeutic resistance, leading to more effective clinical practices.

A growing body of research suggests that the interplay between ferroptosis and cancer is highly diverse and dysregulation of ferroptosis ultimately leads to tumorigenesis and therapy response across different types of tumors (25, 32). Recent studies have demonstrated the crucial role of ferroptosis in alternative resistance in tumor cells (33). For instance, while certain malignant cells exhibit refractory to defined treatments inducing apoptosis or necroptosis, they show increased sensitivity towards ferroptosis, and vice versa. Ferroptosis manifests cytological alterations including reduction or loss of mitochondrial cristae, disruption of the mitochondrial outer membrane, and contraction of the mitochondrial membrane (27). Perturbations in mitochondrial metabolism, including mitochondrial stress response, metabolic reprogramming, and abnormalities in the mitochondrial proteasome, are closely associated with liver cancer development and metastasis (34). The primary function of mitochondria is to generate cellular energy via oxidative phosphorylation (OXPHOS) (35). Mitochondrial defects lead to OXPHOS impairment, mitochondrial dysfunction, and consequently increased ROS production. This dysfunction in ROS clearance has been linked to the progression of liver tumors (35). Thus, it becomes evident that ferroptosis significantly impacts liver tumorigenesis.

Notably, mounting evidence indicates that ferroptosis plays a “double-edge sword” role across a wide spectrum of liver diseases (36). On one hand, inhibiting ferroptosis may counteract the pathophysiological progression of several liver diseases, including alcoholic liver injury, non-alcoholic steatogenic hepatitis, and fibrosis. On the other hand, inducing ferroptosis may limit the emergence of secondary resistance to drugs currently utilized for HCC treatment such as sorafenib. Emerging evidence suggests that iron dysregulation is closely associated with various human diseases, particularly liver disease (37–39). The liver performs a crucial role in regulating iron homeostasis, by coordinating gene regulation for iron transport and storage while maintaining optimal levels through mobilization. Liver dysfunction can disrupt this equilibrium of iron homeostasis, leading to an array of iron-related disorders such as anemia and iron overload (40, 41). Biomarkers of iron toxicity, such as disturbances in iron metabolism, imbalances in amino acid antioxidant systems, and accumulation of lipid peroxidation, are observed at different stages of liver disease (37). Therefore, targeting ferroptosis holds promise for preventing the pathophysiological development of liver diseases (37, 42).

Ferroptosis is closely associated with drug resistance in tumors. Tumor cells develop resistance to existing chemotherapy drugs by increasing intracellular GSH, reducing iron accumulation, inhibiting ROS production, and leveraging various other mechanisms (43). Unfortunately, current cancer treatments fail to adequately address the challenge of drug resistance. Emerging studies have demonstrated the crucial role of ferroptosis in eradicating tumor cells and suppressing tumor growth (44). Drugs targeting ferroptosis exert their clinical effects mainly through the following mechanisms: (1) modulation of antioxidant defense via inhibiting the cystine/glutamate antiporter SLC7A11 and glutathione peroxidase 4 (GPX4); (2) regulation of NRF2 (Nuclear factor erythroid 2-related factor 2)-mediated antioxidant gene expression via p62-Keap1-NRF2 pathway; (3) activation of ferroptotic stimulators via manipulating lysosomes, ferritin, transferrin, and autophagic bodies involved in iron metabolism (44). Previous investigations into drug-induced ferroptosis have laid a comprehensive foundation for future clinical translation of ferroptosis to cancer therapies.

In recent years, accumulating evidence has suggested a clear correlation between the efficacy of immunotherapy and ferroptosis in cancer (45, 46). For instance, the activation of CD8+ T cells by immunotherapy facilitates iron-mediated cell death in cancer cells, thereby enhancing the antitumor potency of immunotherapy (45). Recently, novel therapeutic strategies targeting iron-induced cell death in liver cancer have been identified, and its combination with immune checkpoint blockers warrants further clinical exploration (47, 48).

Sorafenib and lenvatinib represent pivotal first-line therapeutic options for advanced HCC (9). Importantly, Sorafenib is the only anticancer agent capable of inducing ferroptosis in liver cancer patients, significant attention has been focused on elucidating the role of ferroptosis in sorafenib resistance (49, 50). The administration of Sorafenib induces ferroptosis in HCC cells (51). By inhibiting the xCT system’s function, Sorafenib can activate endoplasmic reticulum stress and induce iron-dependent cell death (52). These findings provide novel insights into drug resistance mechanisms in HCC.

The Hippo signalling pathway is a key regulator of tissue growth and regulates cell proliferation, differentiation and migration in organ development (53). Dysregulation of the Hippo pathway results in aberrant cell growth and tumors. The Hippo pathway exerts tumor suppressive effects via its core members, including MST1/2, large tumor suppressor kinase 1/2 (LATS1/2) and the transcriptional coactivator Yes-associated protein (YAP) (54–58). Previous studies have demonstrated that the Hippo-YAP/TAZ pathway is a key driver of ferroptosis in epithelial tumors (59, 60). YAP/TAZ bind to members of the transcription enhancer domain (TEAD) family in the nucleus and drive Hippo target genes such as cysteine-rich, angiogenic inducer 61 (CYR61), connective tissue growth factor (CTGF), etc., ultimately leading to tumorigenesis and tumor recurrence (61, 62). Recently, YAP/TAZ have been identified as novel regulators of SLC7A11 gene expression. YAP/TAZ upregulated SLC7A11 expression and allowed HCC cells to escape sorafenib-induced ferroptosis. Furthermore, YAP/TAZ maintained the protein stability, nuclear localization and transcriptional activity of ATF4, which synergistically induced SLC7A11 expression (63). Immunohistochemical results showed that total YAP staining and nuclear YAP staining were more abundant in HCC tissues than in non-tumor regions (64). In addition, the expression of microchromosome maintenance protein 2 (MCM2) is highly correlated with the expression of YAP in HCC tissues and interference with MCM2 inhibits the Hippo pathway by blocking the entry of YAP into the nucleus, which increases the cells’ resistance to sorafenib (65). In summary, the Hippo/YAP pathway promotes sorafenib-induced ferroptosis resistance.

The leukemia inhibitory factor receptor (LIFR) is frequently down-regulated in HCC. Mechanistic studies have shown that loss of LIFR reduces intracellular iron ion entry by upregulating LCN2 and activating the NF-κb pathway, leading to resistance to sorafenib-induced ferroptosis and promoting liver tumourigenesis (66).

NRF2 is a key regulator of redox balance (67). The p62-Keap1-NRF2 pathway upregulates multiple genes involved in iron and ROS metabolism (Metallothionein-1G[MT-1G], quinone oxidoreductase 1 [NQO1], heme oxygenase-1 [HO1], ferritin heavy chain 1 [FTH1], and ATP binding cassette subfamily C member 5[ABCC5]) plays a central role in protecting HCC cells from ferroptosis (68, 69), which induces resistance to sorafenib. For example, NRF2 induces resistance to sorafenib by upregulating MT-1G, which inhibits lipid oxidation in HCC cells (16). High expression of ABCC5 down-regulates ferroptosis by stabilizing SLC7A11 protein and reducing GPX4 depletion, inhibiting lipid peroxidation, and increasing mitochondrial membrane potential (MMP), thereby promoting the development of sorafenib resistance in HCC cells (17).

In conclusion, the pathway of sorafenib resistance associated with ferroptosis has been extensively studies. Therefore, it is crucial to investigate alterations in PTMs of resistance factors.

## 3 PTMs in HCC tumorigenesis and therapy resistance

Proteins, as key players in living cells, have diverse functions including catalysis, transportation, and structural support (70). While the human genome contains approximately 20000 to 30000 genes (71), the size of the human proteome is expected to exceed 1.8 million proteins due to mRNA alternative splicing and PTMs (72).

PTMs are crucial biochemical reactions that covalently regulate protein conformation, activity, and stability (73). It is estimated that PTMs can occur in 50 to 90% of the body’s proteins (74). PTMs encompass processes such as phosphorylation, acetylation, ubiquitination, methylation, succinylation, and most of which are reversible (70). These modifications intricately govern the activity and stability of target proteins, protein interactions, and intracellular distribution (24). The diverse PTMs on various proteins significantly enhance the flexibility and diversity required for functional regulation in complex life activities. Recent advancements in mass spectrometry techniques have significantly enhanced our ability to identify specific modifications on individual proteins, consequently this allows for a more understanding of the functions and roles of PTMs (71, 72, 75).

Previous studies have demonstrated that multiple PTMs regulate the expression and function of tumor-associated proteins, as well as tumor suppressors (76). Liver cancer similarly conforms to this pattern; thus, numerous novel possibilities for tumor diagnosis and therapeutic targeting have been identified. Currently, research on protein modification in liver cancer primarily focuses on acetylation, methylation, ubiquitination, and phosphorylation (77–79). PTMs play a role in liver cancer proliferation, invasion, and metastasis and therapy resistance. The presence of PTMs not only confers proteins with enhanced functional diversity, but also endows them with the ability to respond to stress rigidly and robustly. Importantly, key players in the regulation of ferroptosis have been identified with PTMs (21).

## 4 PTMs in ferroptosis regulator and key contribution in HCC

**Figure 1:**
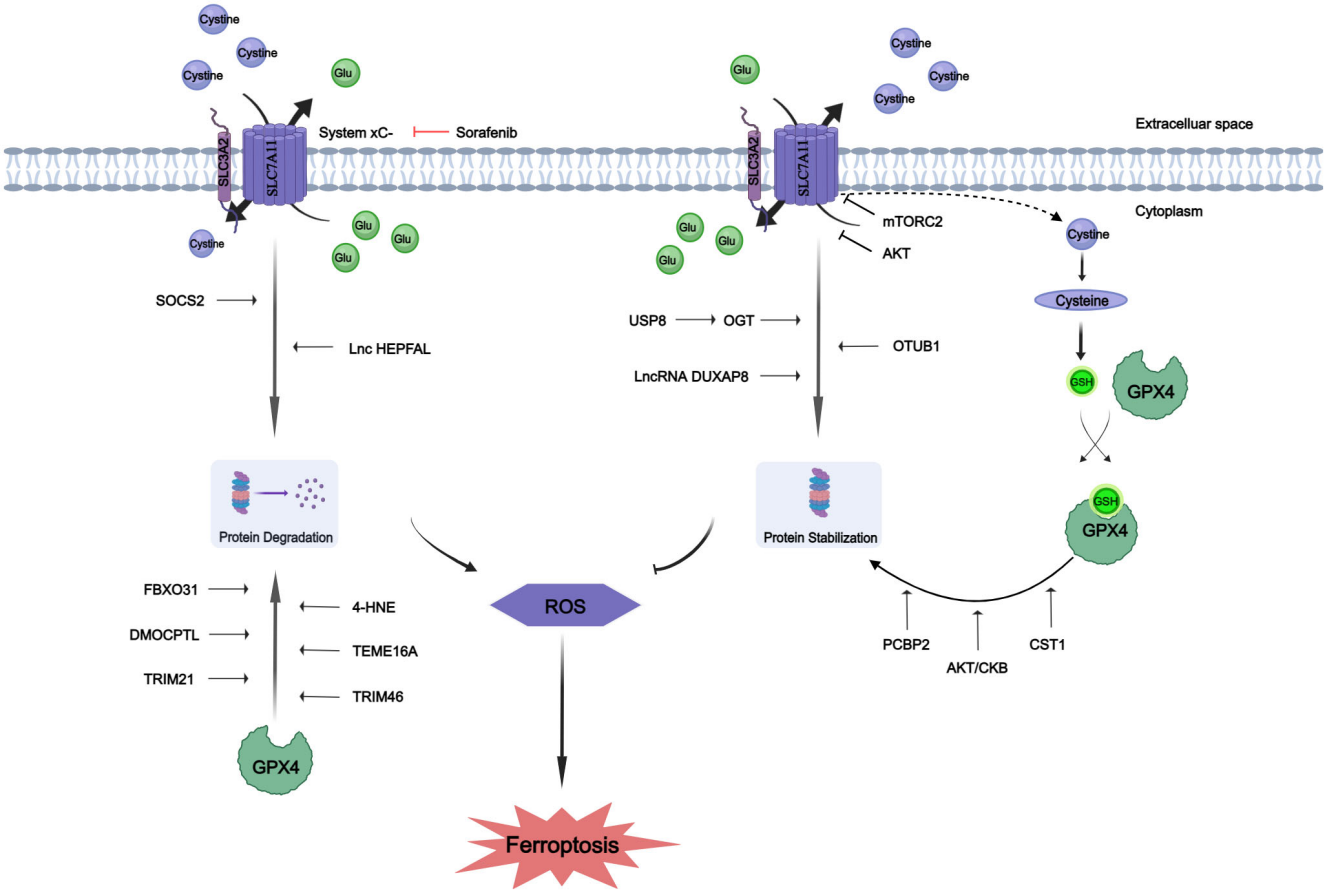
Protein post-translational modifications of SLC7A11 and GPX4. The proteins SLC7A11 and GPX4 underwent post-translational modifications to enhance their stability or promote degradation. Glutamate (Glu).

The intricate biological functions of humans are precisely controlled and catalyzed by proteins, as well as their divergent modified counterparts mediated via PTMs (70). Recent advancements have further emphasized the pivotal role of PTMs in ferroptosis. The xCT system and GPX4 are identified as master regulators of ferroptosis (80). So, we will focus on GPX4 and SLC7A11 in the following discussions. The post-translational mechanisms controlled by xCT may be crucial for tumor cells to rapidly respond to changing environmental conditions (81). GPX4 serves as a key regulatory factor influencing ferroptosis. Recent studies have demonstrated that GPX4 can undergo various PTMs, including ubiquitination, succinylation, and phosphorylation (82–84). Post-translation modification of GPX4 impacts its protein levels and activity, suggesting that manipulating protein PTMs could potentially serve as a therapeutic approach for diseases associated with iron-induced cell death (85). The PTMs of SLC7A11 and GPX4 are described in **Figure 1**.

### 4.1 PTMs in SLC7A11

Lipid peroxidation initiates ferroptosis, while its regulation heavily relies on SLC7A11, an essential constituent of the cystine-glutamate antiporter. A substantial body of previous research has emphasized the importance of transcriptional activity associated with SLC7A11 in the process of ferroptosis; however, our understanding of how SLC7A11 maintains stability in human cancers remains limited beyond its transcriptional regulation (86). Emerging evidence indicates potential involvement of PTMs, including O-GlcNAcylation, phosphorylation and ubiquitination, in modulating SLC7A11 function (87–89). The PTMs of SLC7A11 are summarized in **Table 1**.

**Table 1:** PTMs of SLC7A11.

Key Factors	PTMs	Mechanism and function
**OTUB1**	Ubiquitination	Ubiquitin hydrolase OTUB1 as a key factor in modulating SLC7A11 stability (86)
**OGT**	O-GlcNAcylation	OGT is responsible for the O-GlcNAcylation of SLC7A11, specifically at the Ser26 site of HCC cells (90)
**mTORC2**	Phosphorylation	mTORC2 inhibits the activity of Serine 26 in the n terminal of xCT cytoplasm by phosphorylation (79)
**SOCS2**	Ubiquitination	SOCS2 served as a bridge to transfer the attached ubiquitin to SLC7A11 and promoted K48-linked polyubiquitination degradation of SLC7A11, which ultimately led to the onset of ferroptosis and radiosensitization of HCC (91)
**lncRNA HEPFAL**	Ubiquitination	lncRNA HEPFAL can promote the ubiquitination of SLC7A11 and reduce the stability of the SLC7A11 protein, resulting in decreased expression (92)
**LncRNA DUXAP8**	Palmitoylation	LncRNA DUXAP8 enhances the activity of SLC7A11 by promoting its palmitoylation and inhibiting lysosomal degradation (93)

O-GlcNAcylation exerts its influence on serine and threonine residues of proteins located in the cytoplasm, nucleus, and mitochondria, representing a prevalent, dynamic, and reversible PTM (94, 95). Unlike most other PTMs, O-GlcNAcylation is catalyzed by only two conserved enzymes, namely O-GlcNAc transferase (OGT) and O-GlcNAcase (OGA), for the addition and removal of O-GlcNAc, respectively (96). Aberrant O-GlcNAcylation has been implicated in the malignant properties of cancer cells (53–55). A recent study revealed that inhibition of O-GlcNAcylation leads to mitochondrial fragmentation and enhances mitophagy, thereby providing an additional source of labile iron.This renders the cell more prone to ferroptosis (97). Mechanistic studies have demonstrated that OGT is responsible for the O-GlcNAcylation of SLC7A11, specifically at the Ser26 site in HCC cells. This O-GlcNAcylation plays a critical role in facilitating cystine uptake by SLC7A11 from the extracellular environment. Furthermore, it has been observed that SLC7A11 is regulated by the USP8-OGT axis through O-GlcNAcylation in HCC cells, and this post-translational modification of SLC7A11 is indispensable for its cystine absorption function (90).

The mechanistic targets of the rapamycin protein kinase (mTOR), a highly conserved regulator of cell growth, play a crucial role in linking cell metabolism and growth to various environmental stimuli. As components of mTOR Complex 1 (mTORC1) and mTOR Complex 1 (mTORC2), these targets can promote cell proliferation and survival by phosphorylating AKT (98). Notably, SLC7A11 is regulated by both mTORC1 (99) and mTORC2 (79). The involvement of mTORC2 in cystine uptake and glutathione metabolism by directly phosphorylating xCT establishes a link between alterations in growth factor receptor signaling pathways, amino acid metabolism, and ROS buffering in cancer (79). Studies have demonstrated that mTORC2 interacts with SLC7A11 as a binding partner and phosphorylates Serine 26 within its cytoplasmic region upon stimulation by growth factors, leading to inhibition of its transport activity. Interestingly, another study revealed that AKT, which serves as the primary substrate for mTORC2, directly phosphorylates SLC7A11 at the same site. This AKT-mediated phosphorylation also inhibits cystine transport activity of SLC7A11 (100).

Ubiquitination is a prevalent PTMs that can be reversed by the actions of deubiquitinating enzymes (DUBs), which target numerous oncogenes and tumor suppressor genes involved in cancer progression (101, 102). To date, several DUBs have been identified that regulate ferroptosis by inhibiting xCT system. For instance, ovarian tumor-related proteases (OTU) DUB and ubiquitin aldehyde binding 1 (OTUB1) directly interact with SLC7A11 to stabilize its inhibition of ferroptosis. Additionally, BRCA1-related protein 1 reduces H2Aub occupancy at the promoter region of SLC7A11 leading to decreased expression of SLC7A11, and subsequent inhibition of cystine uptake, resulting in elevated levels of lipid peroxidation (86, 103). It has been observed that SOCS2 (suppressor of cytokine signaling 2) specifically enhances the ubiquitination degradation process of SLC7A11, thereby promoting ferroptosis. Moreover, HCC cells with high expression levels of SOCS2 exhibited significant deletion of SLC7A11 and more pronounced ubiquitination levels for SLC7A11. This suggests that long chain protein B/C interacts with SOCS2 at L162 and C166 to form a complex known as SOCS2/long chain protein B/C, which collectively promotes the ubiquitination degradation of SLC7A11. In both HCC tissues and transplanted tumors, a strong negative correlation between SOCS2 and SLC7A11 was found, subsequently demonstrating that SOCS2 acts as a specific E3 ubiquitin ligase for SLC7A11, thus facilitating ferroptosis by mediating the degradation of SLC7A11 (91).

Additionally, experiments have shown that lncRNA can induce the degradation of SLC7A11 through ubiquitination. Overexpression of lncRNA HEPFAL leads to reduced expression levels of SLC7A11 while inhibiting tumor proliferation and migration. Endogenous expression levels of SLC711 were examined in cells without any difference observed after treatment with the proteasome inhibitor MG-132. However, upon application of CHX, it was confirmed that overexpressed lncRNA HEPFAL led to decreased stability and increased susceptibility to degradation for the SLC7A11 protein, suggesting that lncRNA HEPFAL may facilitate its own degradation through interaction with the ubiquitinated form (92).

The process of palmitoylation plays a crucial role in governing the transportation and functionality of diverse proteins associated with tumors (104, 105), SLC7A11 has been demonstrated to serve as a substrate for the process of palmitoylation (106, 107). There is compelling evidence indicating the direct binding of Double homeobox A pseudogene 8 (DUXAP8) to SLC7A11, and knockdown of DUXAP8 hampers the palmitoylation process of SLC7A11, resulting in its sequestration into lysosomes for degradation. Loss of LncRNA DUXAP8 synergistically enhances sorafenib-induced iron death in HCC. Furthermore, it has been observed that palmitoylation occurs at residue Cys414 in SLC7A11, and DUXAP8 plays a pivotal role in facilitating this modification at the Cys414 site to maintain proper cell membrane localization of SLC7A11. This phenomenon significantly enhances our comprehension of post-translational regulatory mechanisms governing SLC7A11 function and underscores the importance of lncRNA involvement in SLC7A11-mediated metabolic reprogramming and iron-induced cell death in cancer (93).

These collective findings suggest that SLC7A11 can be regulated through various post-translational mechanisms, and modification of SLC7A11 is divergently involved in tumorigenesis and therapy response.

### 4.2 PTMs in GPX4

GPX4 is an enzyme specialized in detoxifying lipid hydroperoxides to lipid alcohols within a membrane environment, relying on the reduction of GSH (108). Insufficient activity of the lipid hydrogen peroxide detoxification pathway leads to ferroptosis, resulting in the oxidation of iron-dependent membrane polyunsaturated fatty acids (PUFA) and accumulation of toxic lipid ROS. Therefore, precise regulation of GPX4 expression and activity plays a critical role in determining cellular ferroptosis (109). GPX4, as one of the most significant antioxidant enzymes, has gained considerable attention over the past decade due to its pivotal regulatory role in cancer, cardiovascular disease, and neuroscience research. The modulation of GPX4 activity has emerged as a prominent topic in current scientific research (85). The PTMs of GPX4 are summarized in **Table 2**.

**Table 2:** GPX4 PTMs.

Key Factors	PTMs	Mechanism and function
**Fumaric acid**	Succinylation	Intracellular accumulation of fumaric acid leads to succinylation of GPX4 at cysteine 93, resulting in both single and double succinylation events, which significantly diminishes the enzymatic activity of GPX4 (82)
**TRIM46**	Ubiquitination	TRIM46 governs the ubiquitination process of GPX4, while high glucose concentration can enhance the ubiquitination of GPX4 (83)
**PCBP2**	Ubiquitination	PCBP2 blocks ubiquitination at the K48 site of GPX4 (110)
**DMOCPTL**	Ubiquitination	DMOCPTL directly binds to the active site of GPX4, leading to the ubiquitination of GPX4 in triple-negative breast cancer cells (111)
**CST1**	Ubiquitination	CST interacts with GPX4, thereby alleviating ubiquitination modification of GPX4 (112)
**FBXO31**	Ubiquitination	FBXO31 up-regulates the ubiquitination process of GPX4 (113)
**TRIM21**	Ubiquitination	GPX4 is a TRIM21 substrate and can be degraded by TRIM21-mediated ubiquitination (114)
**TMEM16A**	Ubiquitination	The interaction between TMEM16A and GPX4 leads to the ubiquitination and degradation of GPX4 (115)
**OTUD5**	Ubiquitination	4-HNE reduces the interaction between OTUD5 and GPX4, and promotes the ubiquitination degradation of GPX4 (116)
**CKB**	Phosphorylation	CKB phosphorylates GPX4, thereby preventing its degradation (84)

Inadequate selenium supplementation and persistent liver inflammation can contribute to the development of HCC. The attack of inflammatory reactive oxygen species on membrane lipids leads to the formation of lipid hydroperoxides, resulting in oxidative damage to the liver. GPX4 plays a crucial role in mitigating this damage by reducing lipid hydroperoxides to their respective hydroxides. The exact role of GPX4 in HCC formation remains unclear; however, it has been demonstrated that GPX4 acts as a tumor suppressor in HCC, particularly when there is significant proliferation. It has been observed that overexpression of GPX4 in HCC cells leads to decreased levels of free radicals, increased GSH levels, and reduced proliferation (117). The protein level of GPX4 is regulated by transcription factors NRF2 or transcription itself. Intracellular supplementation with selenium or glutathione can up-regulate GPX4 activity, while iron allergy inducers such as ML162 and RSL3 can inhibit its activity. These regulatory mechanisms governing GPX4 levels and activity have shown promising potential in preclinical studies for treating diseases associated with iron overload, especially cancer cells. Recent studies have revealed that PTMs like ubiquitination, succinization, phosphorylation, and glycosylation can occur on GPX4. PTMs affecting the protein levels/activity of GPX4 suggest that targeting these processes could be a potential therapeutic approach for iron poisoning-related diseases (85).

Succinate is a non-enzymatic, irreversible protein modification that was discovered in 2006 (118). This post-translational modification is mediated by fumaric acid, an intermediate product of the mitochondrial Krebs cycle. In the absence of enzymes (119, 120), fumaric acid binds to sulfhydryl groups of cysteine residues to form thioether bonds. A study demonstrated that intracellular accumulation of fumaric acid led to succinylation of GPX4 at cysteine 93 (single and double succinylation), resulting in a significant reduction in enzyme activity (82). This study provides evidence for targeting PTMs of GPX4 as a promising therapeutic strategy for diseases associated with iron poisoning.

The regulation of homeostasis in the ubiquitin (Ub) proteasome system (UPS) is potentially crucial for hepatocarcinogenesis. Proteomic analysis revealed ubiquitination of GPX4 at lysine residues 107, 162, and 167 (121, 122). However, these putative ubiquitination sites have not been validated through experimental studies. It has been found that TRIM46 (tripartite motif-containing), a member of E3 Ub ligase family, can regulate the ubiquitination process of GPX4. Moreover, high concentration glucose treatment can up-regulate the ubiquitination level of GPX4 (83). In liver cancer cells, PCBP2 enhances the activity of PSMB5, a major component of proteasomes containing active sites, thereby contributing to TRIB2-induced reduction in overall K48-Ub levels. This results in reduced availability of Ub and prevents K48-ubiquitination of PCBP2, leading to its stabilization. Simultaneously, GPX4’s K48 ubiquitination is blocked, preventing OS-induced damage that could stimulate liver tumorigenesis (110). The compound DMOCPTL, a derivative of the natural product parthenolide (PTL), has been identified as a potential drug for targeting triple negative breast cancer cells (TNBC). It exhibits the ability to induce iron-dependent cell death and apoptosis through GPX4 ubiquitination (111). The interaction between Cystatin (CST) and GPX4 was confirmed through co-immunoprecipitation and mass spectrometry analysis. By recruiting OTUB1, an important deubiquitinase, CST1 mitigates the ubiquitination modification of GPX4, enhances its protein stability, and reduces intracellular ROS, thereby inhibiting iron-induced cell death and promoting gastric cancer metastasis (112). The presence of F-Box Protein 31(FBXO31) enhances the cytotoxic effects of cisplatin in bile duct cancer cells by promoting iron-dependent cell death. This is achieved through the upregulation of ubiquitination processes targeting GPX4, resulting in proteasomal degradation of GPX4 (113). The GPX4 has also been demonstrated to serve as a substrate for Tripartite motif containing 21 (TRIM21), and can undergo degradation through TRIM21-mediated ubiquitination, thereby suggesting that inhibition of TRIM21 could potentially mitigate ferroptosis (114). The interaction between Transmembrane member 16A (TMEM16A) and GPX4 leads to the ubiquitination and degradation of GPX4, thereby promoting ferroptosis (115). The presence of 4-HNE facilitates the carbonylation modification of cysteine residue 93 in GPX4, thereby attenuating the interaction between ovarian tumor (OTU) deubiquitinase 5 (OTUD5) and GPX4, and promoting the ubiquitination-mediated degradation of GPX4 (116).

GPX4 is also subject to phosphorylation. The Zhang group has demonstrated that activation of the insulin-like growth factor 1 receptor (IGF1R) in HCC cells leads to an increase in GPX4 expression, which is dependent on phosphorylated creatine kinase B (CKB) protein kinase activity. CKB phosphorylates GPX4, thereby preventing its degradation and counteracting iron death in HCC cells, ultimately promoting tumor growth (84).

Multiple PTMs of GPX4 have been observed under both physiological and pathophysiological conditions. Targeting PTMs that affect GPX4 could potentially serve as a promising therapeutic strategy for treating diseases. However, research on PTMs in relation to GPX4 is still in its nascent stages, necessitating further exploration.

## 5 Summary and outlook

We provide a comprehensive overview of the pivotal role played by PTMs on key proteins that modulate ferroptosis. Future investigations should focus on determining the functional outputs of PTMs in SLC7A11 and GPX4 in HCC progression and therapy resistance. Moreover, upstream enzymes catalyzing individual modifications are still warrant to be identified. In-depth understanding of PTMs on SLC7A11 and GPX4 will certainly shed new insight into the biology of ferroptosis in pathogenesis and therapy response of HCC and more importantly inspire SLC7A11- and GPX4- based combinatorial therapeutic regimens with improved clinical efficacy.

Currently, limited forms of PTMs have been identified in the ferroptosis pathway due to context dependent assays. With the development of high-throughput facilities and emerging novel toolsets, it is tempting to speculate additional forms of PTMs in divergent ferroptosis proteins on specific residues will be uncovered via unbiased approaches. For instance, it will be of great interest to explore presence and functionals outcomes of emerging PTMs such as methylation and β-Cyclin phosphorylation or crotonylation in ferroptosis proteins. It is envisaged that updated view of global PTMs in therapy response will provide comprehensive understanding of ferroptosis in therapy resistance in HCC and pinpoint potential therapeutic interventions targeting yet to be defined key components of upstream enzymes and ferroptosis-regulating masters.

## Author contributions

YX: Writing – original draft, Writing – review & editing. ZX: Writing – review & editing. RA: Writing – review & editing. ZL: Writing – original draft, Writing – review & editing. FT: Writing – original draft, Writing – review & editing.
